# Supplementary material for: Technical efficiency evaluation of colorectal cancer care for older patients in Dutch hospitals
Source: PLoS One. 2021 Dec 17;16(12):e0260870. doi: 10.1371/journal.pone.0260870 (PMC8682881; doi:10.1371/journal.pone.0260870)
Supplement: S1 File — (DOCX) [file pone.0260870.s001.docx]

**S1 File**

**Original language: Dutch**

1. Datum
2. In welk ziekenhuis bent u werkzaam?
3. Wordt er bij u in het ziekenhuis prehabilitatie toegepast?

- Nee
- Ja, altijd
- Ja, op indicatie

1. Welke van de onderstaande medici en paramedici zijn STANDAARD betrokken in het preoperatieve traject? Meerdere antwoorden mogelijk.

- Diëtiste
- Fysiotherapeut
- Geen van bovenstaande opties

1. Welke van de onderstaande medici en paramedici zijn OP INDICATIE betrokken bij het preoperatieve traject? Meerdere antwoorden mogelijk.

- Diëtiste
- Fysiotherapeut
- Geen van bovenstaande opties

**English**

- 1. Date
  2. In which hospital do you work?
  3. Is prehabilitation applied in the hospital?
- No
- Yes, for every patient
- Yes, by indication

1. Which of the (para)medics below are ALWAYS involved in the preoperative process? Multiple answers possible.

- Dietician
- Physical therapist
- None of the above options

1. Which of the (para)medics below are BY INDICATION involved in the preoperative process? Multiple answers possible.

- Dietician
- Physical therapist
- None of the above options
