# Supplementary material for: Technical efficiency evaluation of colorectal cancer care for older patients in Dutch hospitals
Source: PLoS One. 2021 Dec 17;16(12):e0260870. doi: 10.1371/journal.pone.0260870 (PMC8682881; doi:10.1371/journal.pone.0260870)
Supplement: S2 File — (DOCX) [file pone.0260870.s002.docx]

**S2 File**

DEA was chosen in this study because it can deal with multiple inputs and outputs and needs no assumptions about the distribution between outputs and inputs.[1, 2]

The entire procedure described below is referred to as the Simar & Wilson approach (2007).[3]

**DEA stage 1**

In the first stage of the DEA, the scores of technical efficiency were estimated using the bootstrapped DEA method. The bootstrapped DEA method, resulting in bias-corrected DEA scores, was used since sampling variation and therefore random error was not taken into account in a classical non-parametric DEA analysis. The (semi-parametric) bias-corrected DEA scores are strongly correlated with the uncorrected DEA scores from the classical non-parametric DEA analysis and are mainly important for a valid performance of the parametric regression analysis in stage 2 of the analysis.[4]

An input-oriented model was chosen, meaning that DEA minimizes input for a given level of output. The input-oriented model was used because in this study the input combinations can be altered (involvement of health care professionals), whereas there is no direct control on the number of severe complications (output). It was assumed that all practices were homogeneous in their production technology and assigned the same importance to the inputs and outputs (no weight restrictions were given in the model). Additionally, by assuming that all DMUs were operating at an optimal scale, meaning that an increase in inputs cause the same proportional increase in output, a constant returns to scale (CRS) was chosen.[1]

**The production function**

Input variables: Input is defined here as the average costs of geriatrician, physical therapist and dietician involvement with a patient who is scheduled for colorectal cancer surgery in a period between setting operation indication and admission to the hospital because of tumor resection. Time investment of surgeons and nurses were not taken into account, as no difference between hospitals was assumed.

To indicate involvement, a surgeon and/or a specialized nurse of each participating hospital indicated involvement of physical therapists and dieticians by yes (100%), no (0%) or by indication (only in a selection of patients for whom involvement was thought to be useful). Because it was impossible to quantify the level of involvement in each individual hospital, the involvement ‘by indication’ was quantified based on on clinical data of the Radboud University Medical Center (Nijmegen, the Netherlands). Between 2017-2018 the physical therapist was involved in 5% of the cases in the Radboud University Medical Center and the dietician in 40% of the cases. If there was discrepancy between the surgeon and specialized nurse, the highest involvement scores were chosen. Percentages of involvement of geriatricians were, as mentioned, based on quality indicators published by the Health and Youth Care Inspectorate.[5]

According to the Dutch guidelines for economic health care evaluations, cost prices per patient were calculated by multiplying percentages of involvement with cost prices of geriatricians (€ 91,- per consultation, an average of one consultation was taken), physiotherapist (€33,- per consultation, an average of four consultations was taken) and dietician involvement (€33,- per consultation; average of two consultations was taken).[6]

Output variables: Based on the data obtained from the Dutch Surgical Colorectal Audit the percentages of severe complications in each hospital were calculated. A severe complication was defined as a complication within 90 days after resection with serious consequences: leading to mortality, a surgical reintervention (operative or percutaneous), a postoperative hospital stay of at least 14 days or readmission. As lower values of severe complications represent better quality of care, and DEA usually assumes that more outputs contribute to higher technical efficiency, the percentages of no severe complications were used in the DEA analysis.

**DEA stage 2**

To explain differences in technical efficiency scores, the second stage comprised of bootstrapped truncated regression analysis where estimated technical efficiency scores (bias-corrected DEA scores from the first stage) were regressed on a set of preselected case-mix adjusting and explanatory variables.

**Variables in the regression analyses**

To investigate the relationship between hospital technical efficiency and quality performance and the factors affecting this relationship, bootstrapped truncated regression was employed. The dependent variable was the estimated bias-corrected technical efficiency score of hospitals providing colorectal cancer care surgery.

Case-mix correction was performed by adding the following predefined set of variables to the regression model: 1. ASA score, defined as percentage of patients with a ASA score ≥3, 2. tumor stage, defined as percentage of patients with stage IV colorectal cancer and 3. tumor localization, defined as percentage of patients with colon cancer versus rectal cancer. These case-mix variables were expected to influence preoperative care and postoperative outcome. Age was not taken into account, as mean age between hospitals was expected to be more or less the same because only patients aged ≥ 75 years were included.

In addition, explanatory (independent) variables were selected:

1. Hospital volume: Higher hospital volumes are associated with improved postoperative outcomes.[7]
2. Hospital teaching status: Patients treated in general hospitals have lower severe complications rates in comparison with patients treated in teaching hospitals.[8]
3. Implementation of prehabilitation, categorized in three groups: no, by indication, yes.

**References**

1. Bogetoft P, Otto L. Benchmarking with DEA, SFA, and R.: New York: Springer; 2011.

2. Charnes A, Cooper WW, Rhodes E. Measuring the efficiency of decision making units. European Journal of Operational Research. 1978;2(6):429-44. doi: <https://doi.org/10.1016/0377-2217(78)90138-8>.

3. Simar L, Wilson PW. Estimation and inference in two-stage, semi-parametric models of production processes. Journal of Econometrics. 2007;136(1):31-64. doi: <https://doi.org/10.1016/j.jeconom.2005.07.009>.

4. Simar L, Wilson PW. Sensitivity Analysis of Efficiency Scores: How to Bootstrap in Nonparametric Frontier Models. Management Science. 1998;44(1):49-61.

5. Dutch Health and Youth Care Inspectorate - Basisset MSZ 2017-2018. 2017-2018.

6. Zorginstituut Nederland. Richtlijn voor het uitvoeren van economische

evaluaties in de gezondheidszorg. 2016.

7. Rogers SO, Jr., Wolf RE, Zaslavsky AM, Wright WE, Ayanian JZ. Relation of surgeon and hospital volume to processes and outcomes of colorectal cancer surgery. Annals of surgery. 2006;244(6):1003-11. doi: 10.1097/01.sla.0000231759.10432.a7. PubMed PMID: 17122626.

8. van Groningen JT, Eddes EH, Fabry HFJ, van Tilburg MWA, van Nieuwenhoven EJ, Snel Y, et al. Hospital Teaching Status and Patients' Outcomes After Colon Cancer Surgery. World journal of surgery. 2018;42(10):3372-80. doi: 10.1007/s00268-018-4580-3. PubMed PMID: 29572565.
