# Supplementary material for: Technical efficiency evaluation of colorectal cancer care for older patients in Dutch hospitals
Source: PLoS One. 2021 Dec 17;16(12):e0260870. doi: 10.1371/journal.pone.0260870 (PMC8682881; doi:10.1371/journal.pone.0260870)
Supplement: S1 Table — (DOCX) [file pone.0260870.s003.docx]

| **Data source** | **Description** | **Variables** | **Definition variables** |
| --- | --- | --- | --- |
| Questionnaires | Surveys sent by e-mail via Castors EDC.[1]  Send to surgeons and specialized nurses.  Detailed questionnaire is added in S2. | Involvement of physical therapists  Involvement of dietician  Clinicians’ judgement on prehabilitation implementation | Is physical therapist involved in preoperative care: yes/ no/ by indication  Is dietician involved in preoperative care: yes/ no/ by indication  Is prehabilitation applied in the hospital: yes/ no/ by indication  Yes = 100% of patients  No = 0% of patients  By indication = only in a selection of patients for whom involvement was thought to be useful |
| Quality indicators[2] | On hospital level, the percentage of patients ≥70 years who had surgery for colorectal cancer (years 2017+2018).  On hospital level, the percentage of frail older patients ≥70 years which is assessed by a geriatrician (years 2017+2018).  Published by the Health and Youth Care Inspectorate  Available from: <https://www.dhd.nl/producten-diensten/omniq/Paginas/Databestanden-Basisset-MSZ.aspx> | Involvement of geriatricians | % frail older patients ≥70 years which is assessed by a geriatrician  % patients ≥70 years who had surgery for colorectal cancer  To guarantee anonymity of hospitals, three categories have been made:   1. involvement in < 15% of patients, 2. involvement in 15-25% of patients 3. involvement in >25% of patients |
| Dutch Surgical Colorectal Audit [3] | Nationwide, population based database including informationof patients who undergo a resection of a primary [colorectal carcinoma](https://www.sciencedirect.com/topics/medicine-and-dentistry/colorectal-carcinoma) in the Netherlands (patient, tumor, diagnostic, procedural and outcome data).  Data was requested on hospital level for patients ≥75 years, between 2017-2018.  Available upon request: <https://dica.nl/dcra/onderzoek>. | Descriptive variables  Practice size  Percentage of patients without severe complications | Age, gender, ASA, CCI, tumor localization (colon/rectum), tumor stage, resection type, yes/no neoadjuvant treatment  Number of treated patients between 2017-2018  A severe complication was defined as a complication within 90 days after resection with serious consequences: leading to mortality, a surgical reintervention (operative or percutaneous), a postoperative hospital stay of at least 14 days or readmission. |

**S1 Table**

**References**

1. Castor EDC. Castor Electronic Data Capture 2019 [August 28, 2019]. Available from: <https://castoredc.com>.

2. Dutch Health and Youth Care Inspectorate - Basisset MSZ 2017-2018. 2017-2018. Available from: <https://www.dhd.nl/producten-diensten/omniq/Paginas/Databestanden-Basisset-MSZ.aspx>

3. Van Leersum NJ, Snijders HS, Henneman D, Kolfschoten NE, Gooiker GA, ten Berge MG, et al. The Dutch Surgical Colorectal Audit. European Journal of Surgical Oncology (EJSO). 2013;39(10):1063-70. doi: <https://doi.org/10.1016/j.ejso.2013.05.008>.
