# Supplementary material for: Technical efficiency evaluation of colorectal cancer care for older patients in Dutch hospitals
Source: PLoS One. 2021 Dec 17;16(12):e0260870. doi: 10.1371/journal.pone.0260870 (PMC8682881; doi:10.1371/journal.pone.0260870)
Supplement: S2 Table — (DOCX) [file pone.0260870.s004.docx]

**S2 Table**

| **Hospital number** | **Respondent** | **Is prehabilitation applied in the hospital?** | **Which of the (para)medics below are ALWAYS involved in the preoperative process?** | | | **Which of the (para)medics below are BY INDICATION involved in the preoperative process?** | | |
| --- | --- | --- | --- | --- | --- | --- | --- | --- |
|  |  |  | **Dietician** | **Physical therapist** | **None of the options** | **Dietician** | **Physical therapist** | **None of the options** |
| **1** | Surgeon | NA | NA | NA | NA | NA | NA | NA |
|  | Specialized nurse | Yes, by indication |  |  | X | X | X |  |
| **2** | Surgeon | No |  |  | X | X | X |  |
|  | Specialized nurse | Yes, by indication |  |  | X | X |  |  |
| **3** | Surgeon | No |  |  | X | X | X |  |
|  | Specialized nurse | No |  |  | X | X |  |  |
| **4** | Surgeon | No |  |  | X |  |  | X |
|  | Specialized nurse | No |  |  | X | X | X |  |
| **5** | Surgeon | No |  |  | X | X |  |  |
|  | Specialized nurse | No |  |  | X | X | X |  |
| **6** | Surgeon | Yes, by indication | X | X |  |  |  | X |
|  | Specialized nurse | Yes, for every patient |  |  | X |  |  | X |
| **7** | Surgeon | NA | NA | NA | NA | NA | NA | NA |
|  | Specialized nurse | No | X | X |  |  |  | X |
| **8** | Surgeon | No |  | X |  | X |  |  |
|  | Specialized nurse | No | X | X |  |  |  | X |
| **9** | Surgeon | No |  | X |  |  |  | X |
|  | Specialized nurse | Yes, for every patient |  |  | X |  |  | X |
| **10** | Surgeon | Yes, by indication |  |  | X | X | X |  |
|  | Specialized nurse | No |  |  | X | X |  |  |
| **11** | Surgeon | NA | NA | NA | NA | NA | NA | NA |
|  | Specialized nurse | Yes, by indication |  |  | X | X | X |  |
| **12** | Surgeon | No | X |  |  |  |  | X |
|  | Specialized nurse | Yes, for every patient | X |  |  |  |  | X |
| **13** | Surgeon | No |  |  | X | X | X |  |
|  | Specialized nurse | Yes, for every patient |  | X |  | X |  |  |
| **14** | Surgeon | Yes, by indication |  |  | X | X | X |  |
|  | Specialized nurse | Yes, by indication |  |  | X | X | X |  |
| **15** | Surgeon | Yes, by indication | X |  |  |  | X |  |
|  | Specialized nurse | NA | NA | NA | NA | NA | NA | NA |
| **16** | Surgeon | Yes, by indication |  |  | X |  |  | X |
|  | Specialized nurse | Yes, by indication |  | X |  | X |  |  |
| **17** | Surgeon | No |  |  | X | X |  |  |
|  | Specialized nurse | NA | NA | NA | NA | NA | NA | NA |
| **18** | Surgeon | Yes, for every patient |  |  | X | X |  |  |
|  | Specialized nurse | No |  |  | X | X |  |  |
| **19** | Surgeon | NA | NA | NA | NA | NA | NA | NA |
|  | Specialized nurse | Yes, for every patient |  |  | X | X | X |  |
| **20** | Surgeon | No |  |  | X | X |  |  |
|  | Specialized nurse | NA | NA | NA | NA | NA | NA | NA |
| **21** | Surgeon | No |  |  | X |  |  | X |
|  | Specialized nurse | NA | NA | NA | NA | NA | NA | NA |
| **22** | Surgeon | Yes, for every patient | X | X |  |  |  |  |
|  | Specialized nurse | Yes, for every patient |  | X |  | X |  |  |
| **23** | Surgeon | No |  |  | X | X | X |  |
|  | Specialized nurse | Yes, by indication |  |  | X | X | X |  |
| **24** | Surgeon | No |  |  | X | X | X |  |
|  | Specialized nurse | No |  |  | X |  |  | X |
| **25** | Surgeon | Yes, by indication |  |  | X | X | X |  |
|  | Specialized nurse | NA | NA | NA | NA | NA | NA | NA |

NA= not applicable
