# Supplementary material for: Technical efficiency evaluation of colorectal cancer care for older patients in Dutch hospitals
Source: PLoS One. 2021 Dec 17;16(12):e0260870. doi: 10.1371/journal.pone.0260870 (PMC8682881; doi:10.1371/journal.pone.0260870)
Supplement: S3 Table — (DOCX) [file pone.0260870.s005.docx]

| **Hospital number** | **Hospital size (n)** | **University hospital^a^** | **Mean age (sd)** | **Gender: male (%)** | **ASA ≥ 3 (% )** | **CCI ≥ 1** | **Colon cancer (%)** | **Tumor**  **stage IV (%)** | **Neoadjuvant systemic treatment (%)** | **Laparoscopic approach (%)** | **Severe complications (%)** |
| --- | --- | --- | --- | --- | --- | --- | --- | --- | --- | --- | --- |
| 1 | 14 | Y | 79 (4) | 7 (50%) | 4 (29%) | 3 (21%) | 8 (57%) | 2 (14%) | 3 (21%) | 12(86%) | 4 (29%) |
| 2 | 27 | Y | 79 (4) | 20 (74%) | 15 (56%) | 7 (26%) | 15 (56%) | 6 (22%) | 2 (7%) | 13 (48%) | 8 (30%) |
| 3 | 31 | Y | 79 (3) | 20 (65%) | 14 (45%) | 11 (36%) | 17 (55%) | 7 (23%) | 8 (26%) | 13 (42%) | 12 (39%) |
| 4 | 35 | Y | 79 (4) | 22 (63%) | 10 (29%) | 9 (26%) | 21 (60%) | 3 (9%) | 1 (3%) | 32 (91%) | 8 (23%) |
| 5 | 41 | Y | 78 (4) | 22 (54%) | 15 (37%) | 17 (42%) | 29 (71%) | 3 (7%) | 3 (7%) | 37 (90%) | 9 (22%) |
| 6 | 41 | N | 78 (3) | 22 (54%) | 24 (59%) | 18 (44%) | 30 (73%) | 2 (5%) | 1 (2%) | 36 (88%) | 8 (20%) |
| 7 | 46 | N | 80 (5) | 21 (46%) | 7 (15%) | 11 (24%) | 32 (70%) | 1 (2%) | 1 (2%) | 39 (85%) | 15 (33%) |
| 8 | 64 | N | 79 (5) | 32 (50%) | 29 (45%) | 17 (27%) | 47 (73%) | 5 (8%) | 2 (3%) | 59 (93%) | 26 (41%) |
| 9 | 70 | N | 80 (4) | 37 (53%) | 49 (70%) | 20 (29%) | 46 (66%) | 6 (9%) | 6 (9%) | 64 (92%) | 18 (26%) |
| 10 | 88 | N | 80 (5) | 43 (49%) | 33 (38%) | 24 (27%) | 54 (61%) | 4 (5%) | 5 (6%) | 88 (100%) | 24 (27%) |
| 11 | 98 | N | 81 (4) | 50 (51%) | 35 (36%) | 49 (50%) | 74 (76%) | 11 (11%) | 2 (2%) | 85 (87%) | 20 (20%) |
| 12 | 99 | N | 80 (4) | 54 (55%) | 39 (39%) | 29 (29%) | 70 (71%) | 4 (4%) | 7 (7%) | 86 (87%) | 20 (20%) |
| 13 | 107 | N | 81 (5) | 59 (55%) | 56 (52%) | 36 (34%) | 81 (76%) | 2 (2%) | 1 (1%) | 85 (79%) | 22 (21%) |
| 14 | 112 | N | 79 (4) | 65 (58%) | 49 (44%) | 37 (33%) | 90 (80%) | 2 (2%) | 6 (5%) | 109 (97%) | 20 (18%) |
| 15 | 116 | N | 80 (4) | 62 (53%) | 59 (51%) | 37 (32%) | 83 (72%) | 8 (7%) | 2 (2%) | 115 (99%) | 28 (24%) |
| 16 | 121 | N | 80 (5) | 59 (49%) | 56 (46%) | 57 (47%) | 78 (65%) | 9 (7%) | 2 (2%) | 107 (88%) | 23 (19%) |
| 17 | 124 | N | 80 (4) | 59 (48%) | 56 (45%) | 38 (31%) | 86 (70%) | 4 (3%) | 8 (7%) | 106 (85%) | 41 (33%) |
| 18 | 127 | N | 80 (5) | 38 (30%) | 56 (44%) | 7 (6%) | 91 (72%) | 5 (4%) | 7 (6%) | 120 (94%) | 36 (28%) |
| 19 | 135 | N | 80 (5) | 78 (58%) | 54 (40%) | 45 (33%) | 103 (76%) | 7 (5%) | 3 (2%) | 123 (91%) | 34 (25%) |
| 20 | 136 | N | 80 (4) | 74 (54%) | 61 (45%) | 35 (26%) | 99 (73%) | 5 (4%) | 14 (10%) | 103 (76%) | 28 (21%) |
| 21 | 140 | N | 81 (5) | 71 (51%) | 77 (55%) | 58 (41%) | 101 (72%) | 2 (1%) | 3 (2%) | 127 (91%) | 37 (26%) |
| 22 | 145 | N | 79 (4) | 81 (56%) | 74 (51%) | 40 (28%) | 94 (65%) | 8 (6%) | 7 (5%) | 135 (93%) | 42 (29%) |
| 23 | 163 | N | 80 (5) | 80 (49%) | 45 (28%) | 49 (30%) | 105 (64%) | 16 (10%) | 8 (5%) | 144 (88%) | 26 (16%) |
| 24 | 187 | N | 81 (4) | 85 (46%) | 50 (27%) | 67 (36%) | 131 (70%) | 8 (4%) | 14 (8%) | 178 (95%) | 40 (21%) |
| 25 | 203 | N | 80 (5) | 110 (54%) | 121 (60%) | 66 (33%) | 128 (63%) | 8 (4%) | 10 (5%) | 180 (89%) | 44 (22%) |

**S3 Table**

^a^ N= no, Y= yes
