# Supplementary material for: Technical efficiency evaluation of colorectal cancer care for older patients in Dutch hospitals
Source: PLoS One. 2021 Dec 17;16(12):e0260870. doi: 10.1371/journal.pone.0260870 (PMC8682881; doi:10.1371/journal.pone.0260870)
Supplement: S4 Table — (DOCX) [file pone.0260870.s006.docx]

| **Hospital number** | **Hospital size (n)** | **(extended) Right hemicolectomy (%)** | **(extended) Left hemicolectomy (%)** | **Partial mesorectal excision (%)** | **Total mesorectal excision (%)** | **Abdominoperineal resection (%)** | **Sigmoid resection (%)** | **Other (%)** |
| --- | --- | --- | --- | --- | --- | --- | --- | --- |
| 1 | 14 | 4 (29%) | 2 (14%) | 0 (0%) | 4 (29%) | 1 (7%) | 2 (14%) | 1 (7%) |
| 2 | 27 | 10 (37%) | 2 (7%) | 1 (4%) | 3 (11%) | 7 (26%) | 3 (11%) | 1 (4%) |
| 3 | 31 | 9 (29%) | 3 (10%) | 0 (0%) | 8 (26%) | 5 (16%) | 6 (19%) | 0 (0%) |
| 4 | 35 | 10 (29%) | 2 (6%) | 3 (9%) | 7 (20%) | 2 (6%) | 9 (26%) | 2 (6%) |
| 5 | 41 | 15 (37%) | 0 (0%) | 2 (5%) | 4 (10%) | 6 (15%) | 14 (34%) | 0 (0%) |
| 6 | 41 | 16 (39%) | 4 (10%) | 4 (10%) | 2 (5%) | 3 (7%) | 9 (22%) | 3 (7%) |
| 7 | 46 | 20 (44%) | 6 (13%) | 0 (0%) | 10 (22%) | 1 (2%) | 9 (20%) | 0 (0%) |
| 8 | 64 | 28 (44%) | 8 (13%) | 2 (3%) | 5 (8%) | 6 (9%) | 13 (20%) | 2 (3%) |
| 9 | 70 | 31 (44%) | 8 (11%) | 4 (6%) | 11 (16%) | 4 (6%) | 11 (16%) | 1 (1%) |
| 10 | 88 | 29 (33%) | 9 (10%) | 2 (2%) | 16 (18%) | 4 (5%) | 17 (19%) | 11 (13%) |
| 11 | 98 | 41 (42%) | 6 (6%) | 11 (11%) | 7 (7%) | 3 (3%) | 22 (22%) | 8 (8%) |
| 12 | 99 | 37 (37%) | 5 (5%) | 8 (8%) | 11 (11%) | 8 (8%) | 24 (24%) | 6 (6%) |
| 13 | 107 | 49 (46%) | 7 (7%) | 1 (1%) | 17 (16%) | 2 (2%) | 27 (25%) | 4 (4%) |
| 14 | 112 | 48 (43%) | 8 (7%) | 0 (0%) | 15 (13%) | 0 (0%) | 34 (30%) | 7 (6%) |
| 15 | 116 | 48 (41%) | 14 (12%) | 12 (10%) | 6 (5%) | 2 (2%) | 24 (21%) | 10 (9%) |
| 16 | 121 | 45 (37%) | 9 (7%) | 1 (1%) | 23 (19%) | 7 (6%) | 28 (23%) | 8 (7%) |
| 17 | 124 | 54 (44%) | 9 (7%) | 4 (3%) | 14 (11%) | 13 (11%) | 23 (19%) | 7 (6%) |
| 18 | 127 | 46 (36%) | 9 (7%) | 0 (0%) | 31 (24%) | 6 (5%) | 26 (21%) | 9 (7%) |
| 19 | 135 | 66 (49%) | 11 (8%) | 0 (0%) | 8 (6%) | 15 (11%) | 23 (17%) | 12 (9%) |
| 20 | 136 | 55 (40%) | 15 (11%) | 7 (5%) | 15 (11%) | 10 (7%) | 27 (20%) | 7 (5%) |
| 21 | 140 | 56 (40%) | 11 (8%) | 1 (1%) | 29 (21%) | 4 (3%) | 29 (21%) | 10 (7%) |
| 22 | 145 | 62 (43%) | 14 (10%) | 5 (3%) | 21 (15%) | 8 (6%) | 24 (17%) | 11 (6%) |
| 23 | 163 | 81 (50%) | 14 (9%) | 8 (5%) | 25 (15%) | 11 (7%) | 16 (10%) | 8 (5%) |
| 24 | 187 | 76 (41%) | 14 (8%) | 20 (11%) | 16 (9%) | 17 (9%) | 34 (18%) | 10 (5%) |
| 25 | 203 | 87 (43%) | 15 (7%) | 33 (16%) | 14 (7%) | 11 (5%) | 26 (13%) | 17 (8%) |

**S4 Table**
